# Sulindac has strong antifibrotic effects by suppressing STAT3-related miR-21

**DOI:** 10.1111/jcmm.12506

**Published:** 2015-02-20

**Authors:** Xue Zhou, You-Jie Li, Shu-Yan Gao, Xiao-Zhi Wang, Ping-Yu Wang, Yun-Fei Yan, Shu-Yang Xie, Chang-Jun Lv

**Affiliations:** a Department of Clinical Medicine, Binzhou Medical UniversityYantai, China; b Key Laboratory of Tumour Molecular Biology in Binzhou Medical University, Department of Biochemistry and Molecular Biology, Binzhou Medical UniversityYantai, China; c Department of Respiratory Medicine, Shengli Oilfield Central HospitalDongying, China; d Department of Respiratory Medicine, Affiliated Hospital of Binzhou Medical UniversityBinzhou, China

**Keywords:** pulmonary fibrosis, sulindac, STAT3, IFN-γ, epithelial mesenchymal transition, miRNA

## Abstract

Pulmonary fibrosis (PF) is a disease with an unknown cause and a poor prognosis. In this study, we aimed to explore the pathogenesis of PF and the mechanism of sulindac in attenuating bleomycin (BLM)-induced PF. The rat PF model was induced by BLM and verified through histological studies and hydroxyproline assay. The severity of BLM-induced PF in rats and other effects, such as the extent of the wet lung to bw ratios, thickening of alveolar interval or collagen deposition, was obviously ameliorated in sulindac-treated rat lungs compared with BLM-induced lungs. Sulindac also reversed the epithelial mesenchymal transition (EMT) and inhibited the PF process by restoring the levels of E-cadherin and α-smooth muscle actin (SMA) in A549 cells. Our results further demonstrated that the above effects of sulindac might be related to regulating of interferon gamma (IFN-γ) expression, which further affects signal transducers and activators of transcription 3 (STAT3) and phosphorylated STAT3 (p-STAT3) levels. Moreover, higher miR-21 levels with the decreased E-cadherin and increased α-SMA expressions were found in transforming growth factor-β1-treated A549 cells, which can be reversed by sulindac. Collectively, our results demonstrate that by decreasing IFN-γ-induced STAT3/p-STAT3 expression to down-regulate miR-21, sulindac could significantly reverse EMT in A549 cells and prevent BLM-induced PF.

## Introduction

Pulmonary fibrosis (PF) is the most common chronic, largely untreatable, diffused lung disease 1,2. It is an end-stage disease characterized by abnormal lung tissue remodelling, excessive deposition of collagen, aberrant re-epithelialization and angiogenesis, with a consequent progressive destruction of normal lung tissue, rapid loss of lung function and premature death 3. The specific molecular and cellular mechanisms that contribute to disease progression are still unknown; however, we already know that PF is primarily manifested through epithelial mesenchymal transition (EMT) 4, which is a transdifferentiation process that eventually converts epithelial cells into highly motile mesenchymal cells with increased collagen deposition and other extracellular matrix components in the alveolar epithelial cells (AECs).

MicroRNAs (miRNAs) are emerging as powerful regulators of EMT. A previous study demonstrates that signal transducers and activators of transcription 3 (STAT3)-coordinated Lin-28-let-7-HMGA2 and miR-200-ZEB1 circuits initiate and maintain oncostatin M-driven EMT 5. The interplay of these EMT activators, such as HMGA2 and ZEB1 6–8, represses E-cadherin expression. Interleukin-6, which acts on STAT3 signalling and up-regulates miR-21 in an autocrine manner, reportedly contributes to arsenite-induced EMT 9. The JAK-STAT3 signal pathway is also an important signal transduction pathway that can be activated by different cytokines, such as interferon gamma (IFN-γ) 10. Therefore, STAT3 activation through pro-inflammatory cytokines may be responsible for initiating and maintaining the EMT genetic program by regulating the miRNA expression in some diseases 11.

Previous studies have indicated that sulindac, a non-steroidal anti-inflammatory drug, can inhibit bleomycin (BLM)-induced lung fibrosis by improving the bw, decreasing the collagen deposition in lungs, and ameliorating histological evidence 12. Sulindac can also modulate signalling pathways that are essential for several cellular functions including cell growth, survival, and gene expression, for instance sulindac can down-regulate IFN-γ-induced gene expression 13.

However, whether sulindac has an effect on PF by influencing the STAT3-related signals remains uncertain. Therefore, in this study, rat PF models were induced with BLM to investigate whether sulindac can ameliorate the PF by affecting the STAT3-related signals. Our results demonstrate that sulindac can mitigate PF by inhibiting endogenous IFN-γ and IFN-γ-induced STAT3-related factors, thereby providing experimental targets for clinical PF treatment.

## Materials and methods

### Animals and ethics

An intratracheal BLM (5 mg/kg) has been reported to result in marked lung fibrosis, and it is the most commonly used and referenced model to stimulate PF 14,15^.^ Therefore, we established a BLM-induced PF model in the present study.

Healthy eight-week old female SD rats, weighing 180–220 g, were purchased from the Yantai Green Leaf Experimental Animal Center. All animal experimental procedures in this study were conducted in compliance with Institutional Animal Care and Use Committee guidelines and approved by the Committee on the Ethics of Animal Experiments of Binzhou Medical University. The animals were randomized into six groups. In the 0-day group, the rats were untreated. Rats were given a single intratracheal instillation of BLM (5 mg/kg; Sigma-Aldrich, St. Louis, MO, USA), and were killed at the 7th day post-treatment (7-day group), at the 14th day post-treatment (14-day group), and at the 28th day post-treatment (28-day group). In the sulindac group, the BLM instilled rats were treated with sulindac (20 mg/kg/day; purity ≥98%; Sigma-Aldrich) starting at 14th day after the intratracheal BLM instillation and with daily intragastric administration for 14 days. In the sham group, the rats were given intratracheal instillation of a physiological saline solution with the same volume, and were killed at the 28th day post-treatment. The bw was measured weekly. The severity of lung inflammation and fibrosis, as well as the effect of sulindac on PF were assessed through histological evaluation, collagen delineation and the pathological score, as previously reported.

### Cell culture

About A549 cells (human type II AECs) were obtained from the Shanghai Institute of Cell Biology. The cells were cultured in RPMI-1640 (Hyclone, USA) supplemented with 10% foetal bovine serum (FBS), 100 U/ml penicillin and 100 μg/ml streptomycin at 37°C with 5% CO_2_ atmosphere. Confluent cell cultures were maintained in serum-free RPMI-1640 containing 0.1% FBS for 24 hrs prior to stimulation with cytokines. In the sulindac treatment experiments, A549 cells were pre-incubated with exogenous transforming growth factor (TGF)-β1 (5 ng/ml, Sigma-Aldrich) for 24 hrs before treating with sulindac, after they were co-incubated for 48 hrs. For the positive control (ALK5)-SB431542 hydrate (5 nmol/ml, Sigma-Aldrich), as a specific inhibitor of the TGF-β1 receptor type I kinase, was used to pre-treat the cells for 30 min. before treating and co-incubating with TGF-β1 for 72 hrs. In the miRNA experiments, transient (Table S1) transfection of miR-21 was performed with Lipofectamine 2000 according to the manufacturer's instructions (Invitrogen, USA). The cells were then co-cultured in serum-free RPMI-1640 containing FBS for 48 hrs. Afterwards, the cells were collected and the morphological changes in A549 cells were observed under an inverted microscope. All experiments were conducted in triplicate.

### Haematoxylin and eosin staining and immunohistochemistry

After sacrifice, part of the lung tissue was fixed in 4% neutral formalin. It was routinely dehydrated, embedded and then sectioned for haematoxylin and eosin staining. Immunohistochemistry analysis was performed to detect protein expression using the avidin-biotin-peroxidase method. Briefly, the sections were incubated with the primary antibodies rabbit antirat IFN-γ antibody (dilution 1:200; Beijing Biosynthesis Biotechnology Co., Ltd, China) at 4°C over night. After incubating with the appropriate biotinylated secondary antibody (1:5000; Bei Jing Zhong Shan-Golden Bridge Technology Co., Ltd., China) and avidin-biotin-peroxidase complex, a characteristic brown colour was obtained and recorded as a positive result.

### Western blot

As previously described 16, protein (35 μg) was loaded in each lane of a SDS2 polyacrylamide gel, and then transferred to a nitrocellulose membrane. The membranes were blocked with 5% non-fat milk in TBST [50 mmol/L Tris-HCl (pH 7.6), 150 mmol/l NaCl, and 0.1% Tween-20] for 2 hrs at room temperature. Subsequently, the membranes were incubated with a rabbit or mouse antirat antibody [IFN-γ, 1:200, Beijing Biosynthesis Biotechnology Co., Ltd; STAT3, 1:500, Bioworld Technology, Inc, USA; p-STAT3, 1:500, Bioworld Technology, Inc; E-cadherin, 1;400, Santa Cruz Biotechnology, Inc, USA; α-smooth muscle actin (SMA), 1:500, Bioworld Technology, Inc] in blocking buffer at 4°C overnight. After washing with TBST, the membranes were incubated with a horseradish peroxidase (HRP)-conjugated goat antirabbit secondary antibodies (1:5000, Bei Jing Zhong Shan-Golden Bridge Technology Co., Ltd.) at room temperature for 1 hr. The membranes were washed with TBST and protein bands were detected using the ECL/ECL plus kit (Boster Immunoleader, China). The proteins were normalized with glyceraldehyde 3-phosphate dehydrogenase (GAPDH; 1:5000, Bioworld Technology, Co., Ltd., China).

### ELISA

Type-I collagen levels in the rat lung tissues were determined using a rat collagen type I (Col I) ELISA kit (R&D Systems, Abingdon, UK) and performed according to the manufacturer's instructions. Briefly, the tissue samples were placed into the microwell plates which have been coated with purified rat collagen I antibody. After incubation, the HRP labelled second antibody was combined, and then TMB was added. The absorbance of each plate was measured using a wavelength at 450 nm. The collagen I concentration in the samples was then analysed by comparing the optical density (OD) values of the samples with the standard curve.

### Hydroxyproline assay

Hydroxyproline assay was performed to assess collagen synthesis. Briefly, rats were anesthetized and lung tissues were dried and hydrolysed at 95°C for 5 hrs. The acid hydrolysates and standards were added into 1.5 ml tubes, along with the same volume of citric/acetate buffer (citric acid, sodium acetate, sodium hydroxide, glacial acetic acid and propanol, pH 6.0) and chloramine T solution (chloramine T dissolved in Milli-Q water).The tubes were incubated at room temperature for 10 min. and Ehrlich's solution was added to the tubes, and incubated at 60°C for 15 min. The absorbance (OD values at 550 nm) of the reaction product was measured. The hydroxyproline content was quantified and expressed as μg/mg of lung weight.

### Histological studies

Tissue sections were stained with haematoxylin and eosin and Masson trichrome for histopathological examination to examine the fibrotic degree. Each of the randomly selected microscopic fields per section was analysed. All assessments were blindly performed.

### Quantitative real-time polymerase chain reaction (qRT-PCR)

The miRNAs were extracted using a Mirvana™ miRNA kit (Ambion, USA). Poly (A) was added using a poly(A) polymerase (Ambion). The cDNA was synthesized using the RT primer: 5-AACATGTACAGTCCATGGATGd (T)30N(A,G,C or T)-3. The forward primer of miR-21 used to amplify the miRNAs was 5′-AGCTTATCAGACTGATGTTGACTG-3′. The reverse primer was 5′-AACATGTACAGTCCATGGATG-3′. Then, qRT-PCRs were performed with SuperTaq polymerase (Takara) following the manufacturer's instructions. The miRNA expression was assessed using the RG3000 system (Corbett Research) with the Quantitect SYBR-Green kit (Qiagen). Initial denaturation was performed at 95°C for 3 min., followed by 40 cycles of 95°C for 20 sec., 53°C annealing for 20 sec., and extension at 72°C for 20 sec. The fluorescence was measured at 585 nm at each extension step of 72°C. The melting curve data were collected to check the PCR specificity. Human 5S rRNA was used as the control. All experiments were performed in triplicate.

### Immunofluorescence analysis

The cells were fixed with 4% paraformaldehyde in PBS, permeabilized with 0.5% Triton X-100 in PBS, and labelled with E-cadherin-specific antibodies (1:100; Santa Cruz Biotechnology, Inc.), α-SMA-specific antibody (1:100; Bioworld Technology, Inc, USA), and Alexa Fluor 488 donkey antirabbit IgG (H+L) and Alexa Fluor 594 donkey antimouse IgG (H+L) (Molecular Probes, USA). Fluorescence images were captured under an immunofluorescence microscope (Leica DFC500, Germany).

### Statistical analysis

Each analysed parameter was expressed as mean ± SD. anova and a factorial design variance analysis was performed. The statistical differences between the groups were determined using independent samples *t*-test. All data analyses were performed with SPSS for Windows version 13.0. All statistical tests were two-sided and probability values of 0.05 or less were considered statistically significant. All experiments were performed in triplicate.

## Results

### Sulindac attenuates BLM-induced PF in rat

Bleomycin can cause PF as a rare side effect from cancers therapy 17. It can cause lung damage through direct DNA strand breakage and generate free radicals that induce oxidative stress 18. The intratracheal instillation of BLM in the rat model is advantageous because it is simple and only requires a short time before its effect is observed. The wet lung weight is an indicator of lung inflammation or fibrosis 19. Our results showed that the BLM-induced rats had reduced bws and increased per cent relative lung weights compared with saline control rats (sham) after 28 days because of severe tissue damage, which were ameliorated in sulindac-treated rats (Table1).

**Table 1:** Body weight and lung-to-bw ratio (mean ± SD, *n* = 3)

Treatment	Days	bw (g)	Lung-to-bw ratio (%)
BLM	0	190±2.65	1.53±0.03
BLM	7	208±2.00*	1.43±0.02*
BLM	14	218±1.00*	1.32±0.03*
BLM	28	227±2.64*	1.15 ±0.03*
Saline	28	280±2.65†	0.71±0.04†
Sulindac	28	245±1.73†	1.02±0.03†

* *P* < 0.05 compared with the 0 day controls.

† *P* < 0.05 compared with the BLM-treated rats at 28 days.

**Figure 1:**
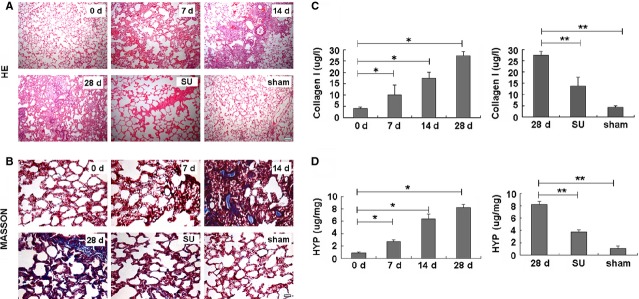
Pathological changes in PF of sulindac-treated rat. (A) Haematoxylin and eosin; scale bars = 200 μm. (B) Masson's trichrome stain for collagen and elastin evaluation; scale bars = 200 μm. In BLM-untreated rats at 0 day, the lung tissues had clear structures without noticeable pathological changes. At 7 days, inflammatory cell infiltration appeared. From 14 to 28 days, although inflammation gradually eased, fibrosis aggravated gradually with noticeably increased collagen deposition and alveolar septa. Most alveolar structures were destroyed. In the sulindac-treated (20 mg/kg/day) PF tissues, the changes from alveolitis to fibrosis were attenuated compared with that in BLM-induced PF. Inflammation and fibrosis were also less serious compared with those in BLM (5 mg/kg)-induced PF. (C) Type-I collagen analysis. Collagen I levels in BLM-treated lung tissues on 28 days increased six folds compared with those in untreated or saline-treated control (sham) lung tissues. Collagen I levels were evidently reduced in sulindac-treated PF tissues compared with BLM treatment. *P < 0.05 *versus* 0 day. **P < 0.05 *versus* 28 days. (D) Hydroxyproline analysis. BLM-treated PF tissues had much higher levels of hydroxyproline, which could be significantly decreased with sulindac treatment. *P < 0.05 *versus* 0 day. **P < 0.05 *versus* 28 days.

To further study the effect of sulindac on mitigating PF, we applied the most commonly used single endotracheal instillation of BLM (0.5 mg/kg) model of PF in rats. About 20 mg/kg/day sulindac was administrated daily per rat based on previous study 20. The results showed that this sulindac dose could attenuate multiorgans inflammation and fibrosis 12,21. In the BLM-treated lungs, noticeable exudation changes were observed and inflammatory cell infiltration appeared after 7 days. Fibrosis was gradually aggravated with thickening of alveolar septa and extensive collagen deposition after 28 days compared with the control-treated lungs (Fig.1A and B). In addition, sulindac-treated lungs evidently exhibited a decrease in the macrophage infiltration, thickening of alveolar septa, and mild collagen deposition compared with those in the BLM-induced models after 28 days (Fig.1A and B).

As collagen deposition is an important indicator of PF, it can be reflected through the type-I collagen content and the hydroxyproline levels 22. Our results revealed that BLM-induced rat lungs showed significant increase in the type-I collagen deposition (Fig.1C) and hydroxyproline level (Fig.1D) compared with the control treatment. However, lung type-I collagen content and hydroxyproline level decreased in the sulindac-treated lungs compared with those of BLM-induced rats (Fig.1C and D). The above results indicate that sulindac attenuates BLM-induced PF.

### Sulindac decreases IFN-γ expression in PF

**Figure 2:**
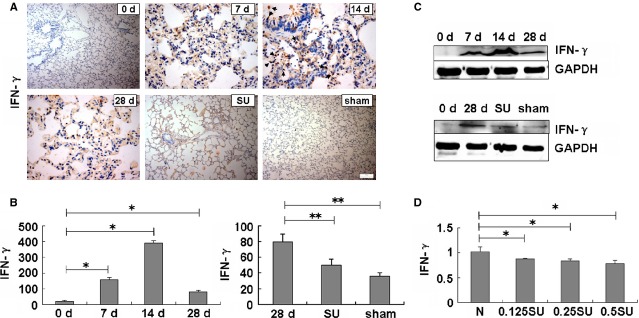
Interferon-γ expression was suppressed by sulindac. (A) Immunohistochemical analysis; scale bars = 100 μm. (B) Integrated optical density analysis. IFN-γ expression obviously increased in the BLM-untreated tissues, especially in 14 days BLM-treated lungs. However, expression in sulindac-treated tissues was lower compared with the 28 days BLM-untreated lungs. *P < 0.05 *versus* 0 day. **P < 0.05 *versus* 28 days. (C) Western blot analysis showed that the IFN-γ level evidently decreased in sulindac-treated rats compared with those in 28 days BLM-treated lungs. GAPDH was used as a control. *n* = 3 replicates. (D) ELISA analysis of A549 cell supernatant. IFN-γ expression was decreased in sulindac-treated cells compared with negative controls. *n* = 3 replicates.

Interferon-γ, a cytokine produced by NK cells and type I helper T cells, increases surface Fas expression in human type 2 AECs as well as activation and apoptosis 23, which has a deleterious effect on PF. After BLM exposure for 28 days, IFN-γ staining evidently increased in BLM-treated lung tissues (7, 14 and 28 days BLM-treated rats), as shown by the immunohistochemistry results (Fig.2A and B). Western blot assay further showed that the IFN-γ level gradually increased with time in the BLM-induced lung tissues compared with the control treatment. The IFN-γ expression peaked after 14 days, and was higher than the saline-treated control after 28 days (Fig.2C, Fig. S1A).

A previous report has shown that sulindac treatment leads to a reduced expression of pro-inflammatory cytokines such as IFN-γ compared with the controls 24. Therefore, to explore the sulindac mechanism for relieving of pulmonary inflammation and fibrosis, we investigated its effect on IFN-γ expression in BLM-induced PF. Our results revealed that sulindac could significantly reduce IFN-γ expression in lung tissues compared with saline-treated rats (Fig.2A–C). Moreover, to further test the effects of sulindac on IFN-γ expression, A549 cells were treated with sulindac at different concentrations *in vitro*. After incubating with sulindac for 48 hrs, we collected the supernatant of the cells for ELISA analysis. Our results showed that the IFN-γ content gradually decreased with increasing concentration of exogenous sulindac (from 0.125 to 0.5 mmol/l, Fig.2D), but higher concentration (more than 0.125 mmol/l) of sulindac has cytotoxicity to inhibit cell growth. So we treated A549 cells with 0.125 mmol/l sulindac in the following study. Altogether, the above results demonstrated that sulindac could evidently suppress IFN-γ activation in BLM-treated PF or A549 cells.

### Sulindac inhibits IFN-γ-induced STAT3-related signals

**Figure 3:**
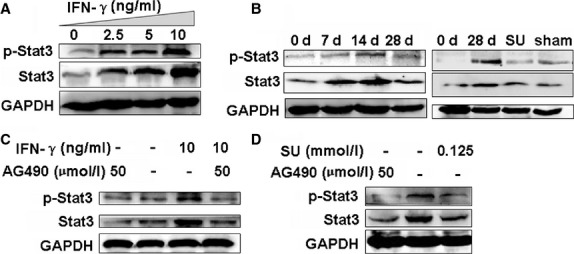
Western blot analysis of STAT3 and p-STAT3 expression. (A) IFN-γ induced STAT3 and p-STAT3 expression in concentration-dependent manner, especially in 10 ng/ml IFN-γ-treated A549 cells. *n* = 3 replicates. (B) STAT3 and p-STAT3 expression gradually and evidently increased in BLM-treated lungs compared with untreated controls. However, STAT3 and p-STAT3 expressions in sulindac-treated tissues were significantly lower compared with that of the 28 days BLM-treated lungs. *n* = 3 replicates. (C) AG490 (50 μmol/l) blocked 10 ng/ml IFN-γ-induced p-STAT3 in A549 cells. *n* = 3 replicates. (D) The expression of STAT3 and p-STAT3 was attenuated by sulindac (0.125 mmol/l) in A549 cells, which was similar to the effect of AG490 (50 μmol/l) treatment. *n* = 3 replicates.

Interferon-γ can activate intracelluar JAK/STAT in various diseases processes 10. We also found that the levels of STAT3 and p-STAT3 were increased in IFN-γ-treated A549 cells (Fig.3A), supporting that IFN-γ can activate STAT3-related signals. Although the specific role of STAT3 in fibrosis is still unclear, studies on the liver, skin, kidney, and the present study on BLM-induced rats have illustrated its importance in fibrotic pathogenesis 25. Our results showed that the STAT3 and p-STAT3 expressions gradually increased in the BLM-induced lung tissues of rats after 7 days, peaked at 14 days, and then subsequently decreased. However, the STAT3 expression was still higher than that in saline-treated controls after 28 days (Fig.3B), demonstrating that STAT3 has important functions in the pathogenesis of BLM-induced fibrosis.

We therefore proposed to investigate whether sulindac can ameliorate PF by regulating JAK/STAT3 activation in PF. Indeed, the STAT3 and p-STAT3 levels in the sulindac-treated lung tissues were significantly lower than those in only BLM-treated rats after 28 days (Fig.3B). The above results demonstrated that IFN-γ may activate STAT3, and sulindac exerted an anti-PF effect by inhibiting the STAT3 and p-STAT3 levels.

To further verify the roles of sulindac in regulating the IFN-γ-induced STAT3 activation, we determined whether the overexpression of exogenous sulindac and IFN-γ could have affect STAT3 and p-STAT3 levels *in vitro*. We found that IFN-γ application (10 ng/ml) evidently increases the STAT3 and p-STAT3 expression in A549 cells (Fig.3A). In addition, when A549 cells were treated with the specific JAK2 inhibitor (AG490) to block STAT3 phosphorylation, IFN-γ (10 ng/ml) could not promote STAT3 phosphorylation upon AG490 (50 μmol/l) treatment (Fig.3C). Our results further showed that sulindac (0.125 mmol/l) notably reversed the STAT3 and p-STAT3 expressions compared with the untreated control cells (Fig.3D). The p-STAT3 expression was significantly blocked after A549 cells were treated with AG490 (50 μmol/l) which was the control group (Fig.3D). Overall, these results suggested that sulindac could suppress the IFN-γ-induced STAT3 pathway in PF.

### Sulindac reduces STAT3 pathway-related miR-21 expression

**Figure 4:**
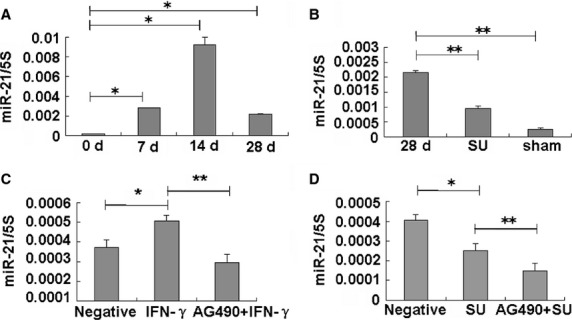
Sulindac reduces miR-21 expression. (A) MiR-21 expression in PF lungs. MiR-21 level increased in the BLM-induced PF tissues, especially at 14 days. *P < 0.05 *versus* 0 day. *n* = 3 replicates. (B) MiR-21 levels were evidently lower in sulindac-treated tissues compared with BLM-treated tissues at 28 days. *n* = 3 replicates. **P < 0.05 *versus* 28 days. (C) IFN-γ (10 ng/ml) could induce the miR-21 expression in A549 cells, which was suppressed by 50 μmol/l AG490. *n* = 3 replicates. *P < 0.05 *versus* negative group. **P < 0.05 *versus*IFN-γ group. (D) Sulindac (0.125 mmol/l) decreased miR-21 expression in A549 cells, which further decreased after AG490 treatment. *n* = 3 replicates. *P < 0.05 *versus* negative group. **P < 0.05 *versus* sulindac group.

MicroRNAs are involved in various diseases, such as cancer, heart, diabetes, and inflammation 26. The let-7d 27 and miR-29 28 down-regulation, and the miR-21 up-regulation 29 contribute to PF. A reporter gene assay has demonstrated that miR-21 overexpression is dependent on STAT3 activation 30. Moreover, the above studies have showed that the STAT3 and p-STAT3 expressions increased in PF. Therefore, we investigated whether miR-21 expression is affected by sulindac in ameliorating the process of PF through the STAT3-related pathway. Our results showed that miR-21 gradually increased in BLM-treated lungs and peaked after 14 days, and was still higher after 28 days than that in untreated or saline-treated (sham) control lungs (Fig.4A). The increased miR-21 level in BLM-treated lungs sharply decreased upon sulindac treatment compared with the saline control (Fig.4B), indicating that miR-21 expression may be regulated by sulindac through the STAT3-related signals.

To further test this proposal, we investigated miR-21 expression in A549 cells treated with sulindac, IFN-γ, or AG490. The results showed that the miR-21 expression was increased in IFN-γ-treated cells after IFN-γ activated STAT3 (Fig.4C). In addition, the higher miR-21 level induced by IFN-γ was blocked by AG490 treatment (Fig.4C), indicating that miR-21 is a downstream miRNA in the STAT3 pathway. Next, we found that miR-21 expression also evidently decreased in the sulindac-treated A549 cells and further decreased after AG490 treatment (Fig.4D). These findings showed that sulindac reduces miR-21 expression by regulating the STAT3 pathway.

### Sulindac prevents the EMT process of A549 cells

**Figure 5:**
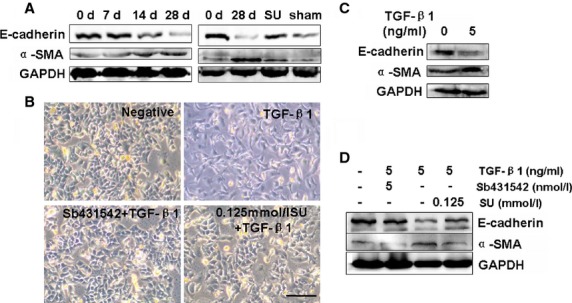
Sulindac prevents TGF-β1-induced EMT in A549 cells. (A) Western blot analysis of E-cadherin and α-SMA expressions in rat lung tissues (*n* = 3 replicates). GAPDH was used as the internal standard. E-cadherin level in lung tissues gradually decreased from 0 to 28 days and α-SMA level in lung tissues peaked at 28 days after BLM treatment. Sulindac (20 mg/kg/day) could evidently maintain E-cadherin and α-SMA expressions. (B) Morphological characteristics; scale bars = 100 μm. TGF-β1 was added into A549 cells for 48 hrs. TGF-β1-treated A549 cells exhibited morphological changes in EMT, converting from their epithelial phenotype into fibroblastic phenotype. Sulindac (0.125 mmol/l) reversed the EMT phenotype of TGF-β1-treated A549 cells. (C) Western blot showed that α-SMA levels increased and E-cadherin was down-regulated in TGF-β1-treated EMT A549 cells. *n* = 3 replicates. (D) Western blot revealed that the levels of α-SMA increased. However, E-cadherin was down-regulated in TGF-β1-treated A549 cells. Sulindac could reverse the EMT-related α-SMA and E-cadherin. *n* = 3 replicates. GAPDH was used as loading controls, *n* = 3 replicates.

Epithelial mesenchymal transition is a process with emerging importance in PF 4. The loss of the epithelial marker E-cadherin and the induction of the mesenchymal marker α-SMA are important markers for the EMT. To further understand the change in these indicators during the EMT in PF, we detected the expression of EMT-related factors (E-cadherin and α-SMA) in BLM-induced lung tissues. The results showed that α-SMA expression markedly increased, whereas E-cadherin gradually decreased in BLM-treated rats from 0 to 28 days (Fig.5A). Sulindac treatment results showed a significant effect of the reverse EMT-related proteins by reducing E-cadherin and increasing the α-SMA levels after 28 days compared with the BLM-treated tissues (Fig.5A). The rat model results showed that BLM would lead to the expression of EMT-related proteins, which can be reversed by sulindac.

To further explore the effect of sulindac on EMT reversal, we established an EMT cell model using TGF-β1. We found that, similar to that in the rat model, TGF-β1 led to overexpression of α-SMA and low E-cadherin level in TGF-β1-treated A549 cells compared with the untreated cells. However, the effect of TGF-β1 would be inhibited by its inhibitor SB431542 (Fig.5B–D). Our results further demonstrated that sulindac treatment (0.125 mmol/l) would lead to a cobblestone-like epithelial morphology (Fig.5B) and result in a better reversal of the TGF-β1-induced E-cadherin and α-SMA expression (Fig.5D).

**Figure 6:**
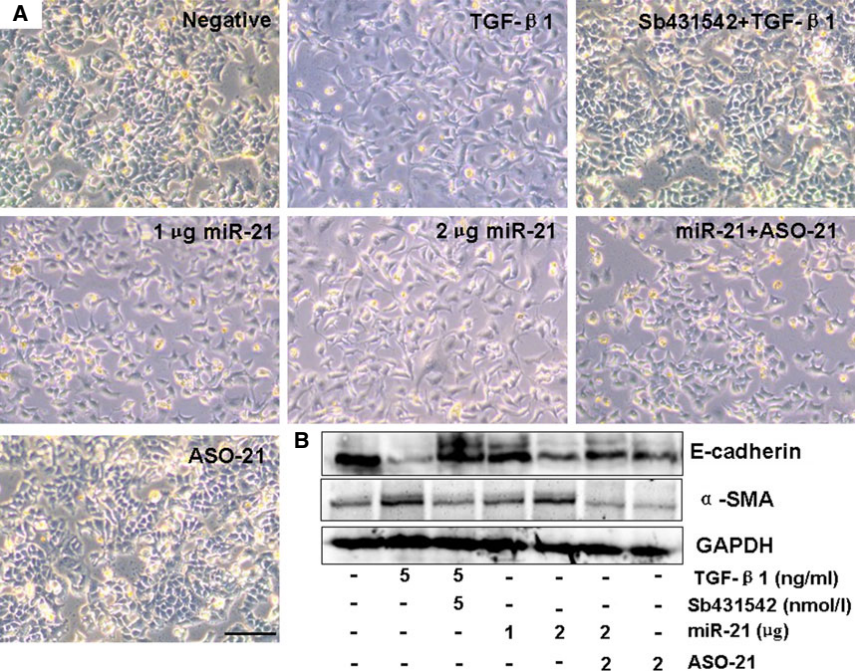
Effect of miR-21 on EMT. (A) Morphological changes; scale bars = 100 μm. The morphology of 2 μg miR-21-treated cells remained fibroblast-like, which was similar to the TGF-β1-induced cells converted from epithelial phenotype into fibroblastic phenotype. The effect of miR-21 was inhibited after 2 μg ASO-21 treatment. (B) Western blot showed that E-cadherin expression decreased and α-SMA expression increased in 2 μg miR-21-treated cells, similar to the TGF-β1-treated cells. *n* = 3 replicates. GAPDH was used as loading controls.

**Figure 7:**
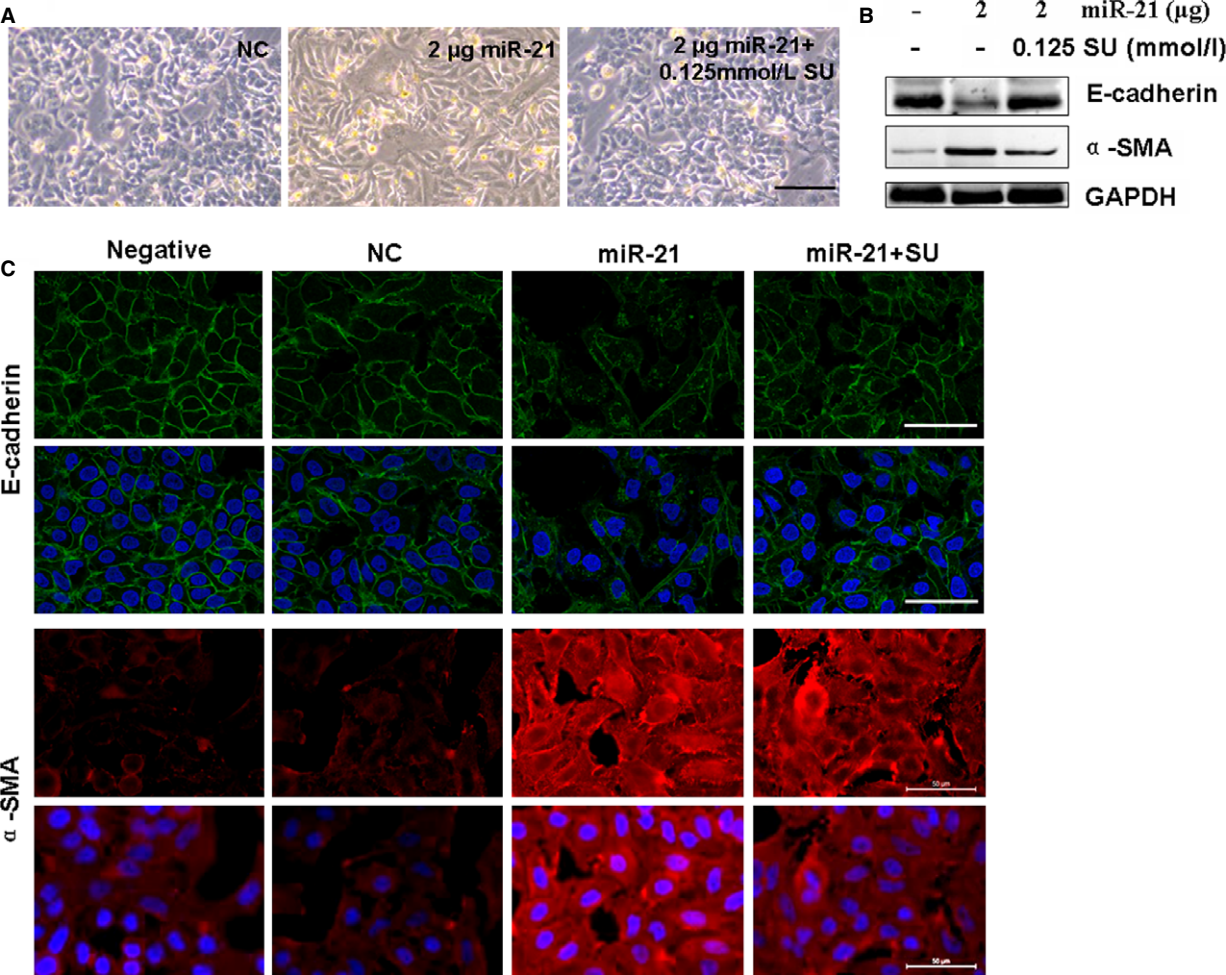
Sulindac suppressed miR-21-induced EMT. (A) Morphological changes; scale bars = 100 μm. The morphology in 2 μg miR-21-treated cells became fibroblast-like cells, which can be reversed by sulindac. (B) Western blot showed that E-cadherin expression decreased and α-SMA expression increased in 2 μg miR-21-treated cells, which can be reversed by sulindac. GAPDH was used as loading controls. *n* = 3 replicates. (C) Cell morphological changes and fluorescence staining. The miR-21-treated A549 cells exhibited morphological changes of EMT, converting from their epithelial phenotype into fibroblastic phenotype, with decreased E-cadherin and increased α-SMA expressions, while sulindac (0.125 mmol/l) reversed the EMT and its marker expression. Red, CY3-labelled E-cadherin; Green, FITC-labelled α-SMA; scale bars = 50 μm.

The above experiments also showed that sulindac reduced miR-21 expression by regulating the STAT3 pathway. An increased miR-21 expression was also reported to be primarily localized in myofibroblasts, mediating the fibrogenic activation of pulmonary fibroblasts and lung fibrosis 29. We then found that 2 μg miR-21 treatment would evidently promote the process of EMT (Fig.6A and B). Western blot assay confirmed the higher α-SMA expression and the lower E-cadherin level in the 2 μg miR-21-transfected A549 cells compared with the control treatment, which was similar to the behaviour of the TGF-β1-treated cells (Fig.6B). Meanwhile, the morphology also maintained fibroblast-like cells in the 2 μg miR-21-treated cultures (Fig.6A). However, the EMT and related factors induced by 2 μg miR-21 can be reversed after miR-21 inhibitor treatment (2 μg ASO-21; Fig.6A and B). Our results illustrated that miR-21 up-regulation can promote EMT in PF. The miR-21-induced EMT would further be ameliorated in the sulindac-treated cells compared with the miR-21 treated cultures (Fig.7A–C). The above results illustrated that sulindac could ameliorate EMT by reducing miR-21 expression.

## Discussion

Previous studies have reported that sulindac, one member of the arylalkanoic acid class, could improve pancreatic and pulmonary inflammation and fibrosis 12,21. In the present study, the mechanism of sulindac in ameliorating PF was investigated. Our results demonstrated that sulindac reversed the TGF-β1-induced EMT in A549 cells and ameliorated the BLM-induced PF in rat lungs by blocking STAT3-realted miR-21 expression. This study provided the evidence of the important role of sulindac in ameliorating EMT and PF by suppressing IFN-γ and STAT3-related factors.

Deposition of excess and abnormal collagen is a characteristic of lung fibrosis 31. Hydroxyproline estimation is a good biochemical index for collagen accumulation and deposition. Therefore, using the midterm administration (sulindac treatment started at 14 days) to mimic the most of the clinical medical records, the effects of sulindac on type-I collagen deposition and hydroxyproline levels in rat lungs were investigated. We found that the type-I collagen and the hydroxyproline levels significantly increased in the BLM-treated lungs, which can be evidently improved by sulindac, suggesting the latter has an antifibrotic effect. This finding was further confirmed through collagen-specific staining and Masson's trichrome staining for collagen deposition in lung biopsy. A remarkable gradual reduction in the bw was also observed in the BLM-treated group, which might be caused by fibrotic progression 32. The increase in the relative weight of the lungs of the BLM-treated rats might be caused by the excessive deposition of collagen 33. Interestingly, we found that sulindac ameliorated the bw and relative weight of the lungs treated with BLM. Lung histopathology results also confirmed the roles of sulindac in relieving PF.

Epithelial mesenchymal transition, a key step in the process of PF, has been described in previous studies 4. A549 cells retain the feature of AEC II cells 34 and can be induced by TGF-β1 to imitate EMT and its important role in PF 2,35,36. In the present study, we found that sulindac could reverse EMT in TGF-β1-induced A549 cells, the mechanism of which might be related to the normalized α-SMA and E-cadherin expressions.

Interferon-γ is one of the central endogenous regulators of immunity and inflammation 37. It increases caspase activation and apoptosis, and also has a deleterious effect in idiopathic PF by up-regualting the surface Fas expression 23. IFN-γ, as a pro-inflammatory cytokines, induces the expression of specific EMT-related genes through STAT3 pathway in Systemic sclerosis, which is a complex disease characterized by vascular alterations, activation of the immune system and tissue fibrosis 38. Similarly, in the present study, we found that the expression of IFN-γ and its related STAT3 was increased in TGF-β1-induced EMT cells (Fig. S3), and the IFN-γ level was also increased in the BLM-induced lung tissues, suggesting the deleterious role of IFN-γ in PF. We also found that sulindac has played protective roles in PF by reducing the endogenous IFN-γ.

JAK-STAT3 signals, an important signal pathway, can be activated by IFN-γ 9. Similarly, in present study, a notably increased expression of STAT3/p-STAT3 was revealed in the IFN-γ-treated A549 cells, and the STAT3/p-STAT3 level is evidently increased with an increase in IFN-γ concentration, confirming that the change in STAT3/p-STAT3 coincides with that of IFN-γ. Moreover, the increased STAT3/p-STAT3 levels, as activated by IFN-γ in A549 cells, were suppressed by the addition of AG490, which may further supported that IFN-γ can mediate the activation of STAT3-related signals. In BLM-induced rat PF model, we found that the expression of IFN-γ and STAT3/p-STAT3 levels peaked at 14 days, and was still much higher than the saline-treated control at 28 days. The reason might be that BLM-induced PF with inflammation at early stage (before 14 days), and fibrosis aggravated gradually with the time elongation at late stage (28 days). Our results revealed that sulindac not only suppressed IFN-γ but also down-regulated STAT3 expression to improve subsequent PF in the rat lungs and in A549 cells, indicating that sulindac can reverse IFN-γ activated STAT3-related signals in PF. STAT3 further regulates the EMT process of several other diseases, resulting in the extinction of E-cadherin and α-SMA expression 39. Our results further revealed that sulindac could reverse PF by regulating STAT3-related E-cadherin/α-SMA expression.

MiR-21, as a STAT3-related factor, contributes to the EMT induced by arsenite 9. The up-regulation of miR-21, also contributes to PF according to previous reports 29, which was supported by our experiments. Notably, increased miR-21 expression in the BLM-induced PF lungs was found. Moreover, miR-21 level remarkably decreased in sulindac-treated rat lungs. E-cadherin down-regulation and α-SMA up-regulation were further observed in miR-21-treated A549 cells, which could be blocked by sulindac effectively. These results demonstrated that sulindac could reverse EMT by regulating STAT3-related miR-21.

In summary, our results demonstrated that sulindac prevents EMT progression and ameliorates PF by reversing IFN-γ-induced STAT3-related miR-21 expression, which highlightes the mechanism of the sulindac application and provides new targets for PF therapy.

## Supporting Information

**Figure S1** Densitometric analysis of Western blots in Figures  2 and 3.

**Figure S2** Densitometric analysis of Western blots in Figures 5–7.

**Figure S3** IFN-γ and its related STAT3 expressions in TGF-β1-induced EMT.

**Table S1** The sequences of chemically synthesized oligos.
