# Supplementary figures and images for: Sulindac has strong antifibrotic effects by suppressing STAT3-related miR-21
Source: J Cell Mol Med. 2015 Feb 20;19(5):1103–13. doi: 10.1111/jcmm.12506 (PMC4420612; doi:10.1111/jcmm.12506)

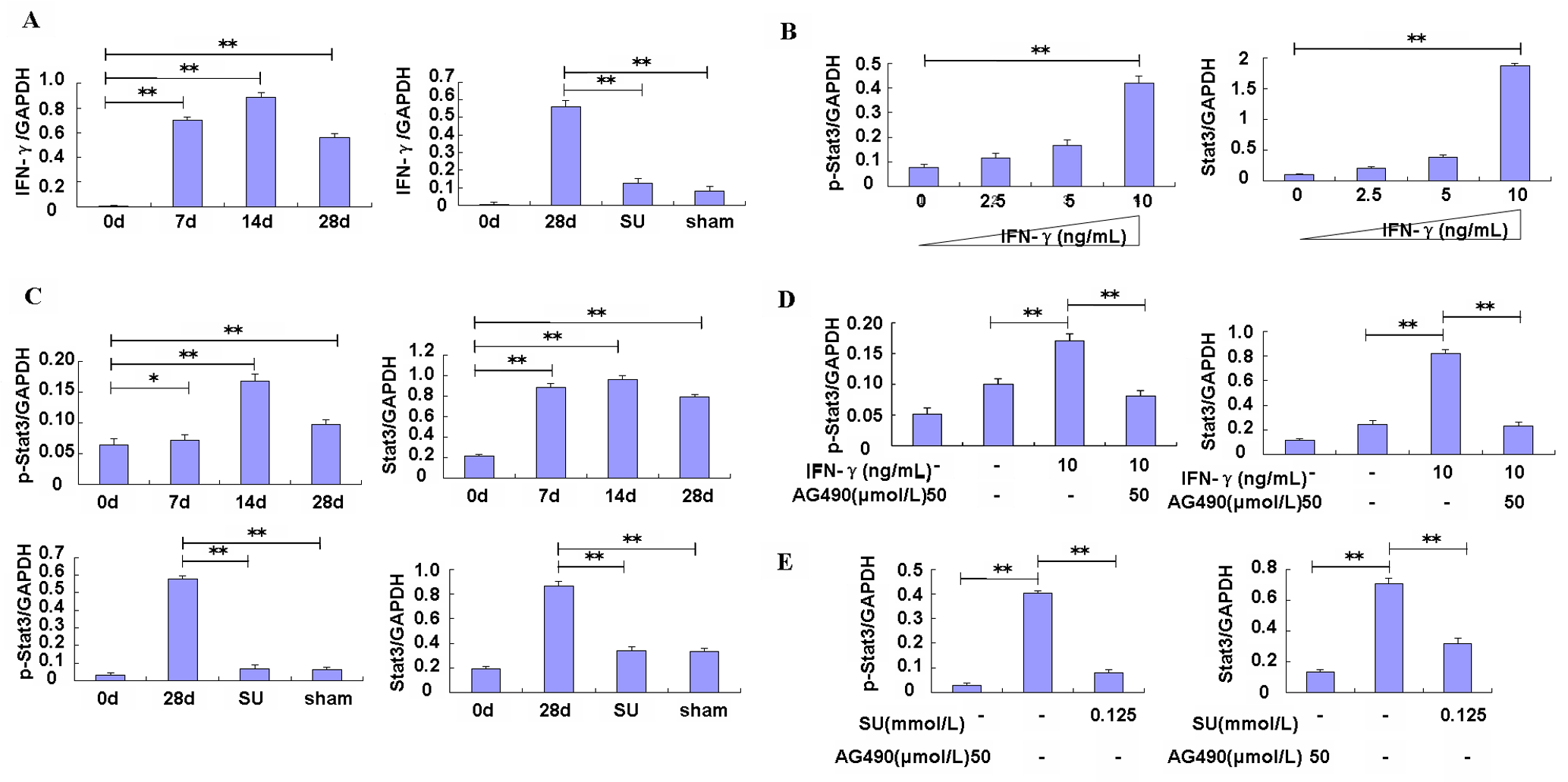

Supplement: Supplementary file 1 [file jcmm0019-1103-sd1.tif]

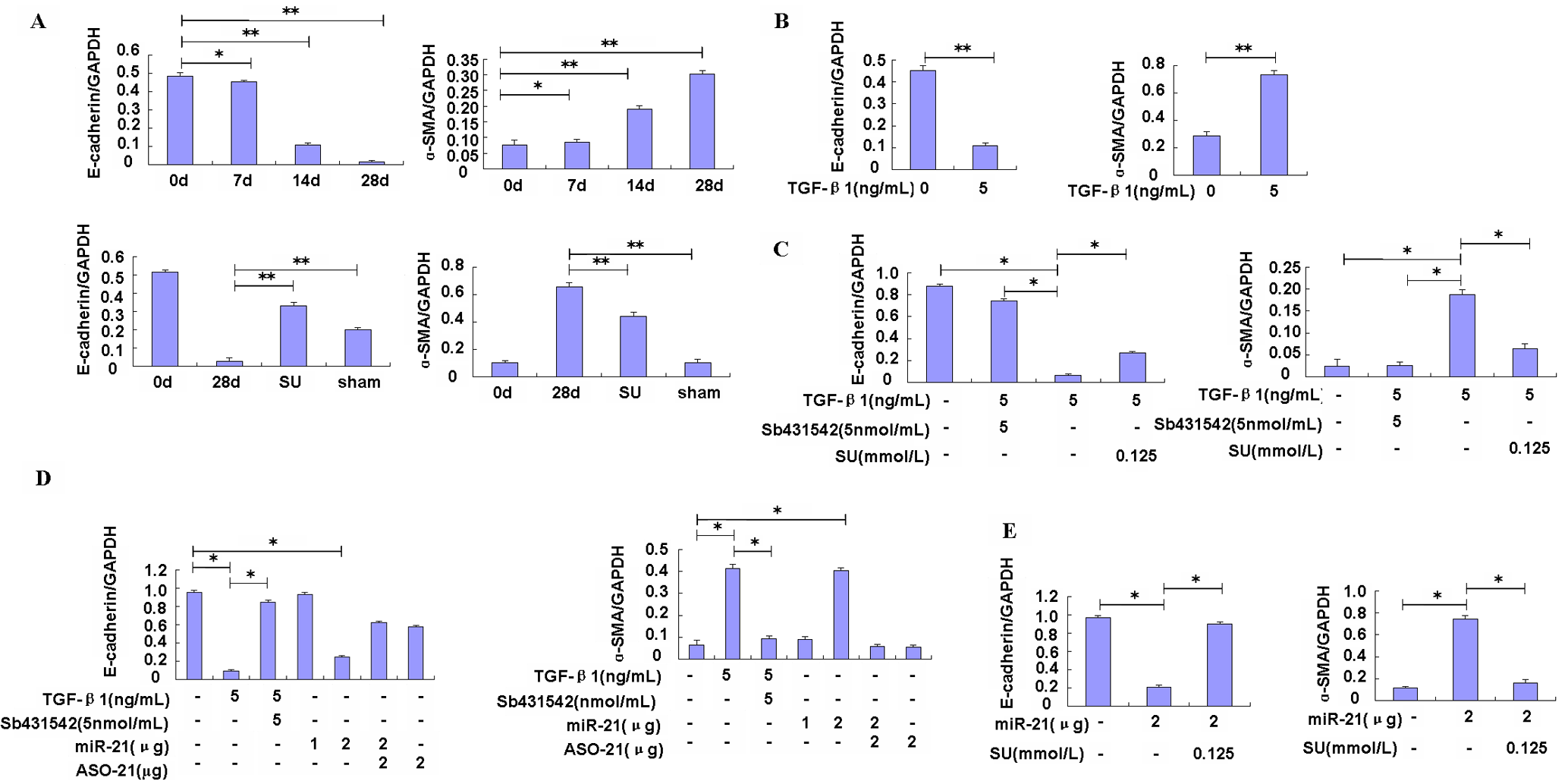

Supplement: Supplementary file 2 [file jcmm0019-1103-sd2.tif]

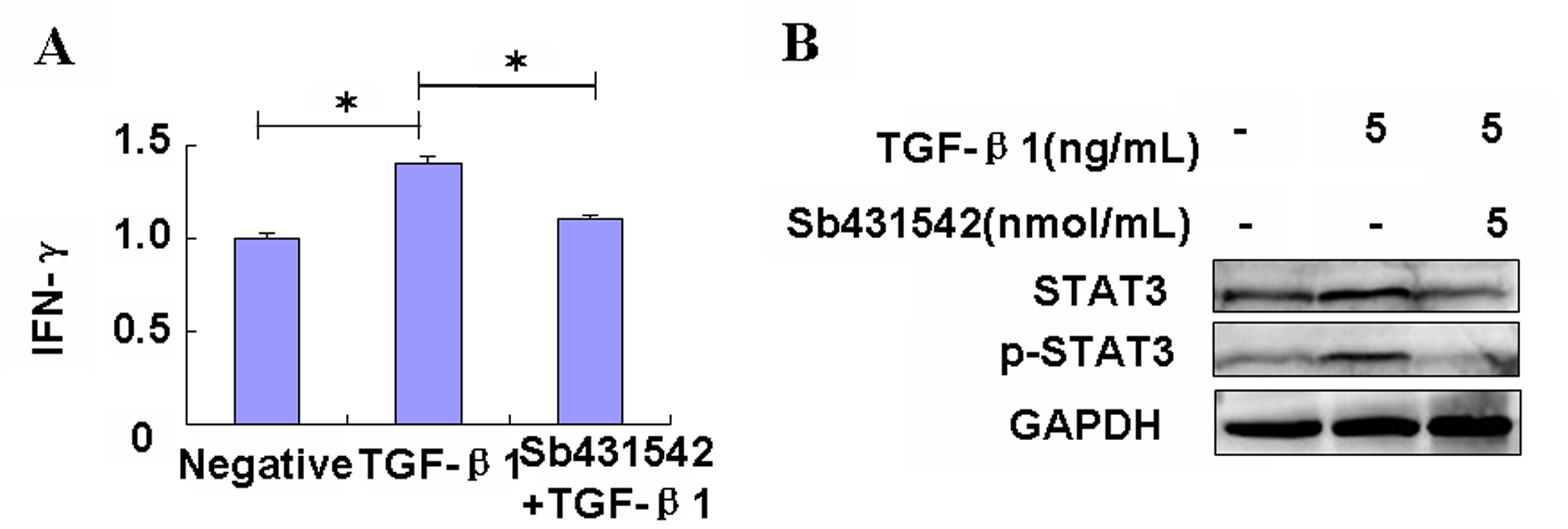

Supplement: Supplementary file 3 [file jcmm0019-1103-sd3.tif]
