# Supplementary material for: Sulindac has strong antifibrotic effects by suppressing STAT3-related miR-21
Source: J Cell Mol Med. 2015 Feb 20;19(5):1103–13. doi: 10.1111/jcmm.12506 (PMC4420612; doi:10.1111/jcmm.12506)
Supplement: Supplementary file 4 [file jcmm0019-1103-sd4.doc]

**Supplemental Table**

**Table S1. The sequences of chemically synthesized oligos**

| **Oligos** | | **Sequence (5'→3')** |
| --- | --- | --- |
| miR-21^a^ | sense | **uagcuuaucagacugauguugauu** |
|  | antisense | **ucaacaucagucugauaagcuauu** |
| ASO-21^b^ | antisense | **ucaacaucagucugauaagcuauu** |
| ^a^The selected miRNAs were chemically synthesized in the form of small interfering RNA (siRNA) duplexes. ^b^Oligos contain 2**'**-OMe modifications. | | |

**Supplemental Figure legends**

**Supplemental Figure S1. Densitometric analysis of Western blots in figures 2 and 3.**

**A:** Densitometric analysis of western blots in figure 2 C. **B-E:** Densitometric analysis of western blots in figure 3 A-D. *P < 0.05 versus control. **P < 0.01 versus control.

**Supplemental Figure S2. Densitometric analysis of Western blots in figures 5, 6 and 7.**

**A-C:** Densitometric analysis of western blots in figure 5A, 5C and 5D. **D:** Densitometric analysis of western blots in figure 6B. **E:** Densitometric analysis of western blots in figure 7B. *P < 0.05 versus control. **P < 0.01 versus control.

**Supplemental Figure S3.** IFN-γ and its related STAT3 expressions in TGF-β1-induced EMT. **A:** The expression of IFN-γ. **B:** The expression of STAT3/p-STAT3. The expression of IFN-r and STAT3/p-STAT3 was found to be enhanced when A549 cells were treated with TGF-β1.
